# High myopia at high altitudes

**DOI:** 10.3389/fphys.2024.1350051

**Published:** 2024-03-08

**Authors:** Ta-Wei Wang, Ming-Kun Huang, Chih-Chun Hsu, Szu-Yang Jo, Yen-Kuang Lin, Chorng-Kuang How, Shih-Fen Tseng, Kong Chung, Ding-Kuo Chien, Wen-Han Chang, Yu-Hui Chiu

**Affiliations:** ^1^ Department of Emergency Medicine, Taoyuan General Hospital, Ministry of Health and Welfare, Taoyuan, Taiwan; ^2^ Department of Emergency Medicine, MacKay Memorial Hospital, Taipei, Taiwan; ^3^ Department of Medicine, MacKay Medical College, New Taipei City, Taiwan; ^4^ Department of Electronic Engineering, National Taipei University of Technology, Taipei, Taiwan; ^5^ Department of Emergency, Hsinchu MacKay Memorial Hospital, Hsinchu, Taiwan; ^6^ Graduate Institute of Athletics and Coaching Science, National Taiwan Sport University, Taoyuan, Taiwan; ^7^ Department of Emergency Medicine, Taipei Veterans General Hospital, Taipei, Taiwan; ^8^ Department of Emergency Medicine, School of Medicine, National Yang Ming Chiao Tung University, Taipei, Taiwan; ^9^ Department of Emergency, School of Medicine, College of Medicine, Taipei Medical University, Taipei, Taiwan

**Keywords:** high myopia, high altitudes, acute mountain sickness, intraocular pressure, optic nerve sheath diameter

## Abstract

**Background:** Optic nerve sheath diameter (ONSD) increases significantly at high altitudes, and is associated with the presence and severity of acute mountain sickness (AMS). Exposure to hypobaria, hypoxia, and coldness when hiking also impacts intraocular pressure (IOP). To date, little is known about ocular physiological responses in trekkers with myopia at high altitudes. This study aimed to determine changes in the ONSD and IOP between participants with and without high myopia (HM) during hiking and to test whether these changes could predict symptoms of AMS.

**Methods:** Nine participants with HM and 18 without HM participated in a 3-day trek of Xue Mountain. The ONSD, IOP, and questionnaires were examined before and during the trek of Xue Mountain.

**Results:** The ONSD values increased significantly in both HM (*p* = 0.005) and non-HM trekkers (*p* = 0.018) at an altitude of 1,700 m. In the HM group, IOP levels were greater than those in the non-HM group (*p* = 0.034) on the first day of trekking (altitude: 3,150 m). No statistically significant difference was observed between the two groups for the values of ONSD. Fractional changes in ONSD at an altitude of 1,700 m were related to the development of AMS (*r*_*pb*_ = 0.448, *p* = 0.019) and the presence of headache symptoms (*r*_*pb*_ = 0.542, *p* = 0.004). The area under the ROC curve for the diagnostic performance of ONSD fractional changes at an altitude of 1,700 m was 0.859 for predicting the development of AMS and 0.803 for predicting the presence of headache symptoms.

**Conclusion:** Analysis of changes in ONSD at moderate altitude could predict AMS symptoms before an ascent to high altitude. Myopia may impact physiological accommodation at high altitudes, and HM trekkers potentially demonstrate suboptimal regulation of aqueous humor in such environments.

## 1 Introduction

High-altitude illness is a potential threat at heights above 2,500 m. In addition to acute mountain sickness (AMS), conditions can progress to life-threatening illness including high-altitude cerebral edema (HACE) and pulmonary edema (HAPE). The prevalence of AMS varies according to the altitude attained, individual susceptibility, rate of ascent, and degree of pre-acclimatization (Luks et al., 2017). Mairer et al. (2009) reported that the prevalence of AMS increased with altitude, from 9.1% at 2,500 m to 38% at 3,500 m. Even in healthy trekkers, exposure to high altitudes will result in several physiological responses, such as hyperventilation, dehydration, changes in oxygen affinity of hemoglobin, and increases in oxidative enzymes (West, 2012). The optic nerve sheath complex comprises the optic nerve sheath, cerebrospinal fluid (CSF), and optic nerve. The optic nerve sheath is an extension of the dura mater and contains subarachnoid CSF. Sonographic measurement of optic nerve sheath diameter (ONSD) has multiple clinical applications, including detection of increased intracranial pressure, optic neuritis, and AMS (Lau et al., 2023). Lau et al. conducted a meta-analysis assessing the ONSD and AMS. The results showed a statistically significant difference in ONSD between participants with and without AMS. However, the heterogeneity among the included studies was high (Tsai et al., 2022). To date, no study has compared participants with and without high myopia (HM) at high altitudes.

Myopia is a common cause of vision loss, and its prevalence continues to increase. In 2010, myopia accounted for 27% of the global population (Nouraeinejad, 2021). In Taiwan, the prevalence of myopia was 95.9% among freshmen at National Taiwan University, approximately one-third of whom had HM in 2005 (Wang et al., 2009). HM is accompanied by structural differences compared to emmetropia. HM commonly manifests as an inherited genetic condition where the eyeball undergoes elongation and excessive growth, causing incoming light to focus in front of the retina. This phenomenon leads to a blurring of vision. In addition to the elongated axial length, pathological changes are also observed in the posterior pole and anterior chamber (Jonas et al., 2019). Changes in the biomechanics and aqueous humor dynamics of myopic eyes may contribute to the susceptibility to intraocular pressure (IOP) and failure to regulate IOP (McMonnies, 2016; Karimi et al., 2022; Li et al., 2022; Machiele et al., 2023). Hence, HM is linked to numerous complications, including macular degeneration, retinal detachment, cataract, open angle glaucoma, and the most severe consequence, blindness.

IOP, which is the fluid pressure inside the eye, is exerted by the aqueous humor in the anterior chamber of the eye. The balance of the aqueous humor is regulated by its production and reabsorption. The aqueous humor is produced by the ciliary body. The majority of the aqueous humor drains through the trabecular meshwork and then into Schlemm’s canal (Machiele et al., 2023). In addition to the physiological regulation of the aqueous humor, IOP is also affected by environmental and individual factors (Kim and Park, 2019; Yang et al., 2019). A recent meta-analysis found that IOP was significantly decreased when trials were conducted at high altitudes (3,000–5,500 m above sea level) but significantly increased when studies were conducted at extreme altitudes (over 5,500 m above sea level). Moreover, IOP decreased when participants were exposed to high altitudes while active, such as during physical activity, trekking, and climbing (Yang et al., 2019).

Until now, little is known about ocular physiological responses, especially in trekkers with myopia at high altitudes. In this study, we aimed to clarify the changes in ONSD and IOP in participants with and without HM when exposed to high altitudes. The secondary aim was to determine the correlation between changes in ONSD and IOP and AMS symptoms at high altitudes.

## 2 Materials and methods

### 2.1 Participants

Twenty-seven healthy participants were enrolled in this prospective study. All participants completed the Xue Mountain trek in March or August 2022. The participants were divided into two groups based on whether they had HM. HM was defined as −6 diopters or more. The Lake Louise AMS scores of the participants, fitness conditions, the use of acetazolamide, IOP, and ONSD were recorded for comparison. All participants lived at an altitude of less than 500 m above sea level, and none of them had been exposed to an altitude above 2,000 m in the 4 months prior to this study. None of the participants had a medical history of hypertension, diabetes, hyperthyroidism, obstructive sleep apnea, glaucoma, ophthalmic surgery, or other ophthalmic diseases. Throughout the entire trek, smoking and the consumption of alcohol were not allowed. All participants provided written informed consent, and the study was approved by the Institutional Review Board of MacKay Memorial Hospital (21MMHIS088e).

### 2.2 Schedule of hiking and timing of examination

**FIGURE 1:**
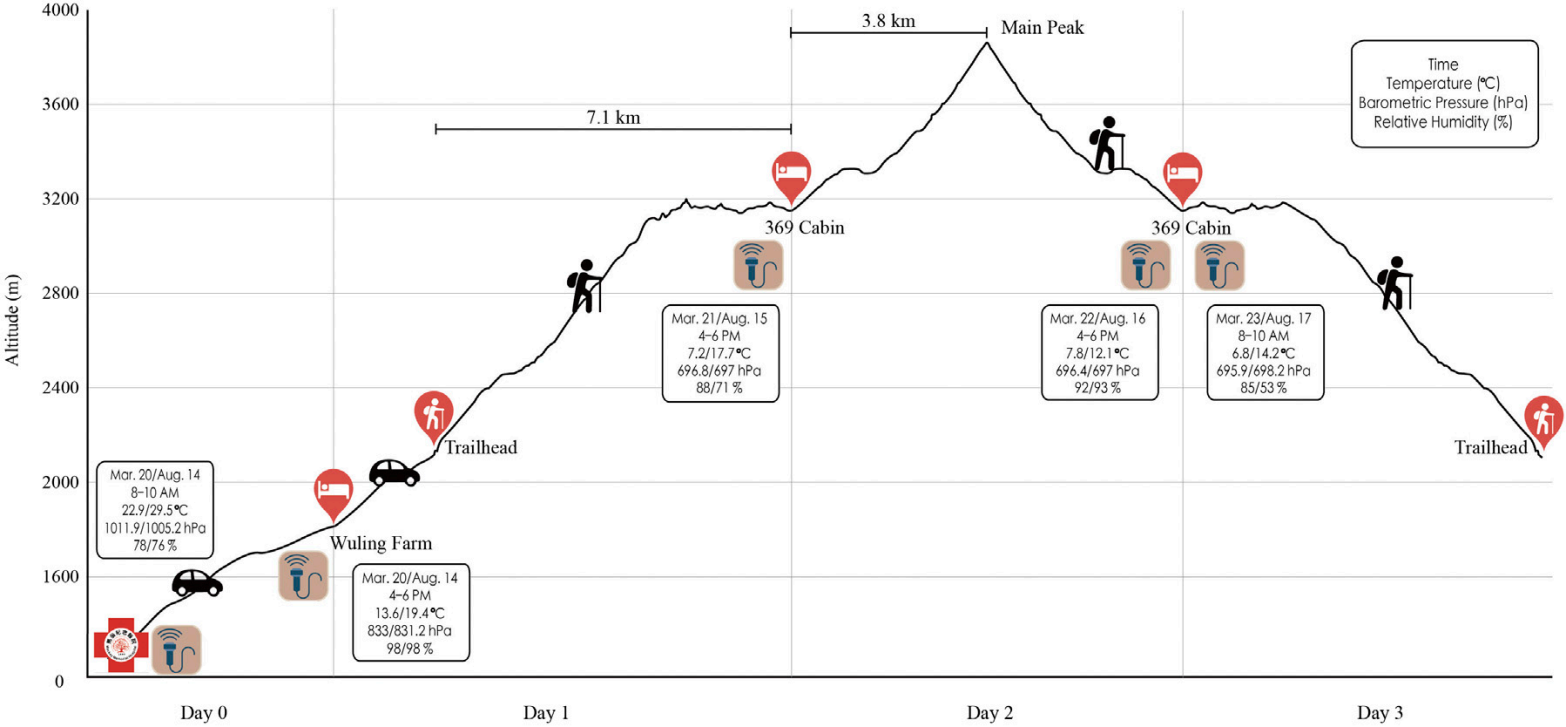
Xue Mountain hiking schedule. Echo icons mean the examining time points. Boxes present both the weather information and the examination time.

This study was designed as a 3-day trek of Xue Mountain (altitude: 2,140–3,886 m), Taiwan (Figure 1). The route began from Taipei City (16 m above sea level) to Wuling Farm (altitude: 1,700 m) by car for 4 h the day before trekking. On the first day of hiking, the participants were driven by car from the lodging location to Xue Mountain trailhead in 20 min (altitude: 2,140 m). Thereafter, they trekked to 369 Cabin (distance: 7.1 km; altitude: 3,150 m) for 8 h. On the second day, they trekked a 3.6 km round trip (summit: 3,886 m, approximately 10 h of walking). On the third day, they descended to the Xue Mountain trailhead.

### 2.3 Ophthalmic examination

Diopter and axial lengths were measured in the lowland 1 week before ascent. Diopter measurements were checked using the KR 8900 (Topcon, Tokyo, Japan). IOP and ONSD were obtained at five different time points: day 0 (8–10 a.m.) in Taipei, day 0 (4–6 p.m.) at Wuling Farm (moderate altitude), day 1 and 2 (4–6 p.m.) in 369 Cabin (high altitude), and day 3 (8–10 a.m.) in 369 Cabin (high altitude). Prior to the ophthalmic examination, participants were advised to rest for a minimum of 15 min. Participants were also instructed to avoid taking acetazolamide within the 8 h preceding the examination. An emergency physician, trained in ultrasound skills through the advanced point-of-care ultrasound (POCUS) courses, conducted ophthalmic ultrasound. Each participant was examined while sitting upright and facing forward. An IC100 (Icare Finland Oy, Finland) rebound tonometer was used to measure the IOP. We used the value of the right eye for statistical analysis. An 8–9 MHz linear probe of the Vscan Air (GE Healthcare, Horten, Norway) was used to measure the ONSD. The ONSD was measured 3 mm behind the eye globe. The ONSD value was obtained by averaging the values of the two eyes.

### 2.4 Vitals recording

Oxygen saturation (SpO_2_) and respiratory rate (RR) were measured using the BeneView T1 (Mindray, China) while the participants quietly sat on a chair after 10 min of rest. The BeneView T1 is a transport monitor approved by the U.S. Food and Drug Administration (size: 10.2 cm × 14.2 cm × 8.1 cm; weight: 0.9 kg). An SpO_2_ probe was placed on the right index finger.

### 2.5 Lake Louise AMS scores

AMS was diagnosed based on the Lake Louise Scoring system (LLSS) for AMS. The LLSS is a self-assessment questionnaire that includes four main symptoms (headache, nausea, dizziness, and fatigue), each rated on a scale of 0–3 (0 = no discomfort, 1 = mild, 2 = moderate, and 3 = severe symptoms). LLSS scores ≥ 3 were considered as symptomatic AMS. Furthermore, we defined mild AMS as 3–5 points, moderate AMS as 6–9 points, and severe AMS as 10–12 points (Roach et al., 2018).

### 2.6 Statistical analysis

All statistical analyses were performed using SPSS version 26 for Windows (Statistical Package for Social Sciences, IBM, Chicago, IL). The Shapiro–Wilk test (*p* > 0.05) and an inspection of the participants’ histograms, normal Q-Q plots, and box plots were used to test the normality of our data. Descriptive results were reported as means ± standard deviations. Fisher exact test was applied for categorical data. An independent samples *t*-test (or Mann–Whitney U test, when appropriate) was used to evaluate the association between participants with and without HM. The repeated measured analysis of variance (ANOVA) was used to evaluate the association between five different time points [day 0 (8–10 a.m.); day 0, 1, and 2 (4–6 p.m.); and day 3 (8–10 a.m.)]. The numerical parameters of IOP and ONSD at 1,700 m and 3,150 m altitudes were compared to the baseline values at ground level using the paired samples *t*-test. The correlations between AMS and fractional changes in IOP and ONSD were calculated using point-biserial correlation. The area under the ROC curve (AUROC), sensitivity, specificity, positive predict value (PPV), and negative predict value (NPV) were applied to obtain the diagnostic performance of ONSD fractional changes. Statistical significance was set at *p* < 0.05.

## 3 Results

The basic characteristics and statistics of participants are described in Table 1. There were 27 participants (37.9 ± 7.1 years; 15 men, 12 women; 9 with HM, 18 without HM) included in this study. Two out of 9 (22.2%) HM trekkers and 2 out of 18 (11.1%) non-HM trekkers had symptoms of AMS (LLSS ≥ 3). Three out of 9 (33.3%) HM trekkers, and four out of 18 (22.2%) non-HM trekkers took acetazolamide for prevention of AMS. HM and non-HM trekkers were similar in all parameters, including age, sex, smoking, snoring frequency, exercise habits, ONSD and IOP values at ground level, AMS diagnosis, acetazolamide use, SpO_2_ and RR at different time points.

**TABLE 1:** Overview of basic characteristics of participants (*n* = 27).

	HM	Non-HM	
	*n* = 9	*n* = 18	*p*-value
Age (years)	38.1 ± 6.8	37.7 ± 7.5	0.897
Gender (male/female)	4/5	11/7	0.431
Diopter of myopia	−7.7 ± 1.9	−2.7 ± 2.0	**0.000**
Smoking (n)	0 (0%)	1 (5.6%)	0.480
Snoring frequency (n)			
None or occasional	8 (88.9%)	9 (50%)	0.091
Frequent	1 (11.1%)	9 (50%)	
Exercise habits (n)			
< 2 times/week	6 (66.7%)	12 (66.7%)	1.000
≥ 2 times/week	3 (33.3%)	6 (33.3%)	
IOP at 16 M (mmHg)	12.9 ± 2.98	12.2 ± 3.26	0.612
ONSD at 16 M (mm)	3.92 ± 0.57	4.01 ± 0.55	0.716
LLSS ≥ 3 (n)	2 (22.2%)	2 (11.1%)	0.582
Prophylactic use of acetazolamide (n)	3 (33.3%)	4 (22.2%)	0.577
SpO2 (%)			
Day 0, 8–10 a.m. (16 M)	97.8 ± 0.7	98.2 ± 1.0	*0.322
Day 0, 4–6 p.m. (1,700 M)	95.1 ± 1.8	95.7 ± 2.0	0.444
Day 1, 4–6 p.m. (3,150 M)	90.0 ± 2.7	88.8 ± 4.4	0.473
Day 2, 4–6 p.m. (3,150 M)	90.2 ± 3.1	88.5 ± 3.8	0.252
Day 3, 8–10 a.m. (3,150 M)	91.2 ± 3.5	90.2 ± 3.7	0.504
RR (bpm)			
Day 0, 8–10 a.m. (16 M)	17.1 ± 4.4	15.3 ± 3.1	0.230
Day 0, 4–6 p.m. (1,700 M)	15.9 ± 2.8	15.1 ± 3.5	0.537
Day 1, 4–6 p.m. (3,150 M)	17.2 ± 2.8	16.8 ± 3.6	0.750
Day 2, 4–6 p.m. (3,150 M)	16.1 ± 2.6	17.4 ± 3.3	0.305
Day 3, 8–10 a.m. (3,150 M)	19.9 ± 3.3	18.2 ± 3.5	0.248

Values presented as mean ± standard deviation and ratio (numbers/total).

*Means *p*-value was obtained from Mann-Whitney U test.

*p*-value < 0.05 is considered as statistically significant.

HM, high myopia; IOP, intraocular pressure; ONSD, optic nerve sheath diameter; LLSS, lake louise scoring system; SpO2, oxygen saturation; M, meters above sea level; RR, respiratory rate; bpm, breaths per minute.

We compared ONSD and IOP between baseline values at ground level and each time point during the trek. Table 2 presents the results of the study. The ONSD values increased significantly in both HM (*p* = 0.005) and non-HM trekkers (*p* = 0.018) on day 0 (4–6 p.m.) at an altitude of 1,700 m. A raise and yet not statistically significant increase in IOP values on day 1 (4–6 p.m.) at an altitude of 3,150 m was noted in HM trekkers (*p* = 0.051). A comparison of the ONSD and IOP values between HM and non-HM trekkers is also shown in Table 2. HM trekkers had statistically higher IOP values than non-HM trekkers (*p* = 0.034) on day 1 (4–6 p.m.) at an altitude of 3,150 m, with no significant difference between the two groups at other time points. There was no statistically difference between the 2 groups for ONSD values at five different time points.

**TABLE 2:** ONSD and IOP during the trek.

	ONSD					IOP				
	HM, *n* = 9		Non-HM, *n* = 18		HM v.s. Non-HM	HM, *n* = 9		Non-HM, *n* = 18		HM v.s. Non-HM
	Mean ± SD	*p*-value	Mean ± SD	*p*-value	*p*-value	Mean ± SD	*p*-value	Mean ± SD	*p*-value	*p*-value
Day 0, 8–10 a.m. (16 M)	3.92 ± 0.57	-	4.01 ± 0.55	-	0.716	12.9 ± 3.0	-	12.2 ± 3.3	-	0.612
Day 0, 4–6 p.m. (1,700 M)	4.32 ± 0.40	**0.005**	4.17 ± 0.50	**0.018**	0.461	13.1 ± 2.5	0.845	11.5 ± 2.7	0.114	0.149
Day 1, 4–6 p.m. (3,150 M)	4.18 ± 0.32	0.233	3.93 ± 0.50	0.431	0.178	15.0 ± 3.0	0.051	12.2 ± 3.0	1.000	**0.034**
Day 2, 4–6 p.m. (3,150 M)	4.20 ± 0.36	0.277	4.00 ± 0.42	0.937	0.225	13.7 ± 2.7	0.592	12.2 ± 3.3	0.934	0.282
Day 3, 8–10 a.m. (3,150 M)	4.33 ± 0.60	0.171	4.07 ± 0.49	0.561	0.227	12.0 ± 2.2	0.437	12.3 ± 3.6	0.942	0.834

Data was shown in mean ± standard deviation (SD). *p*-value < 0.05 is considered as statistically significant.

ONSD, optic nerve sheath diameter; IOP, intraocular pressure; v.s., *versus*; HM, high myopia.; M, meters above sea level.

**FIGURE 2:**
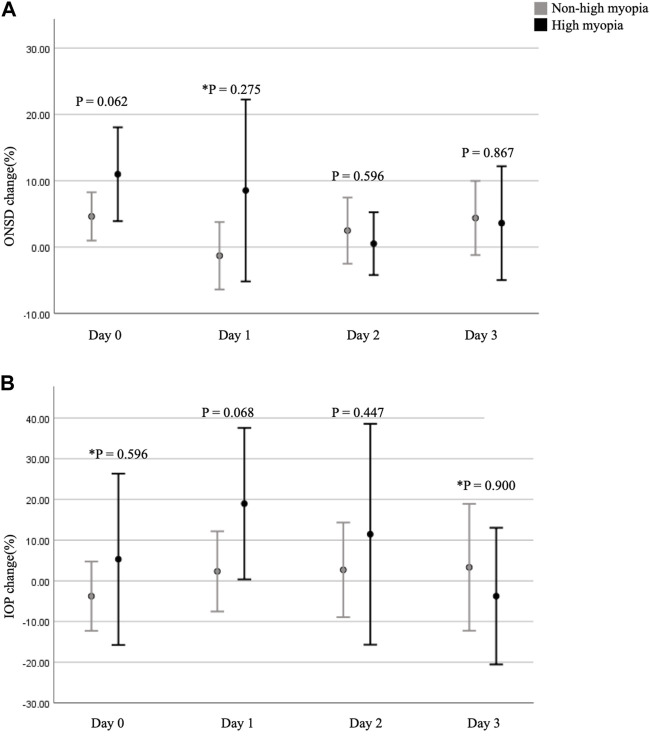
Fractional changes in ONSD and IOP at different time points. **(A)** shows a comparison of the fractional changes in ONSD between the two groups. **(B)** shows a comparison of the fractional changes in IOP between the two groups. *p*-value < 0.05 is considered as statistically significant. * means *p*-value was obtained by Mann-Whitney U test. ONSD, optic nerve sheath diameter; IOP, intraocular pressure.

**FIGURE 3:**
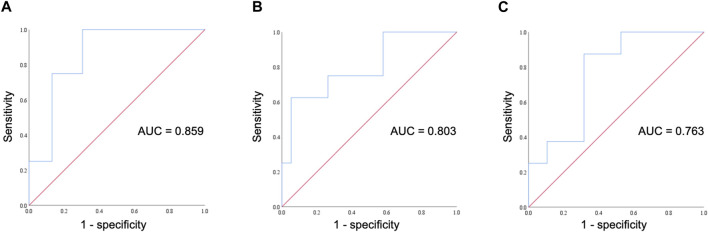
ROC curve for assessing the diagnostic performance of ONSD fractional changes. **(A)** The ROC curve illustrates fractional changes in ONSD on day 0 at an altitude of 1,700 m and its correlation with AMS occurrence on day 2. **(B)** The ROC curve illustrates fractional changes in ONSD on day 0 at an altitude of 1,700 m and its correlation with headache occurrence on day 2. **(C)** The ROC curve illustrates fractional changes in ONSD on day 1 at an altitude of 3,150 m and its correlation with headache occurrence on day 2. ROC, receiver operating characteristic; AUC, area under the curve; ONSD, optic nerve sheath diameter; AMS, acute mountain sickness.

Comparing participants with and without HM, differences in the fractional changes in ONSD and IOP during the trek are shown in Figure 2. The fractional changes in ONSD and IOP are shown as percentages, which were obtained by dividing the differences by the baseline values at ground level. From the ground to 3,150 m, there was no statistically significant difference for the fractional changes in ONSD and IOP values between the two groups. The relationship between trekkers developing AMS or not (scores 3–5 and < 3, respectively) on day 2 and fractional changes in ONSD and IOP at different time points are shown in Table 3. The LLSS on day 2 correlated with fractional changes in ONSD level on day 0 (4–6 p.m.) at an altitude of 1,700 m positively (*r*_*pb*_ = 0.448, 95% confidence interval: 0.082, 0.707, *p* = 0.019). The diagnostic performance of ONSD fractional changes was evaluated through ROC curve analysis (Figure 3A). The AUROC was 0.859 (95% confidence interval, 0.704–1.000). If we employ a cutoff of a 7.315% increase in ONSD fractional changes, the sensitivity and specificity are 100% and 69.6%, respectively. The PPV and NPV are 36.4% and 100%, respectively.

**TABLE 3:** The point biserial correlation of trekkers with LLSS between 3 and 5 (*n* = 4) or < 3 (*n* = 23) on day 2 and the fractional changes in IOP and ONSD at different time-points.

	∆ONSD		∆IOP	
	rpb, [95% CI]	*p*-value	rpb, [95% CI]	*p*-value
Day 0, 4–6 p.m. (1,700 M)	0.448, [0.082, 0.707]	**0.019**	0.125, [−0.267, 0.482]	0.534
Day 1, 4–6 p.m. (3,150 M)	0.274, [−0.118, 0.592]	0.167	−0.153, [−0.503, 0.241]	0.445
Day 2, 4–6 p.m. (3,150 M)	0.209, [−0.185, 0.545]	0.295	0.285, [−0.106, 0.6]	0.150
Day 3, 8–10 a.m. (3,150 M)	−0.016, [−0.393, 0.366]	0.938	−0.042, [−0.415, 0.343]	0.836

*p*-value < 0.05 is considered as statistically significant.

LLSS, lake louise scoring system; ONSD, optic nerve sheath diameter; IOP, intraocular pressure; ∆, (ascending value–ground value)/ground value; *r*_*pb*_, point-biserial correlation coefficient; 95% CI, 95% confidence interval; M, meters above sea level.

As headache and nausea are important symptoms of AMS, we conducted a further evaluation to explore the correlation between trekkers experiencing headaches or nausea on day 2 and fractional changes in ONSD and IOP at different time points. There was a positive correlation between the occurrence of headaches on day 2 and fractional changes in ONSD levels on day 0 (4–6 p.m.) at an altitude of 1,700 m (*r*_*pb*_ = 0.542, 95% confidence interval: 0.205, 0.764, *p* = 0.004), as well as on day 1 (4–6 p.m.) at an altitude of 3,150 m (*r*_*pb*_ = 0.527, 95% confidence interval: 0.184, 0.755, *p* = 0.005). The diagnostic effectiveness of ONSD fractional changes on day 0 at an altitude of 1,700 m (Figure 3B) and on day 1 at an altitude of 3,150 m (Figure 3C) was evaluated through ROC curve analysis. Applying a cutoff of a 2.82% increase in ONSD fractional changes on day 0 at an altitude of 17,00 m results in a sensitivity and specificity of 100% and 42.1%, respectively. Alternatively, using a cutoff of a 4.31% decrease in ONSD fractional changes on day 1 at an altitude of 3,150 m yields a sensitivity of 100% and a specificity of 47.4% for detecting the occurrence of headaches. We observed no discernible association between nausea and fractional changes in both ONSD and IOP at all time points.

## 4 Discussion

Approximately fifteen percent of the participants experienced mild AMS during the trek. This study demonstrated fractional changes in ONSD at an altitude of 1,700 m correlated with AMS positively in a 4-day itinerary. IOP levels in the HM group were greater than those in the non-HM group on the first day of trekking at an altitude of 3,150 m. Increased IOP values in HM participants ascending from the low elevated ground to a high altitude of 3,150 m, though which was not significant.

### 4.1 Changes in ONSD between groups with and without HM exposure in high-altitude environment

The clinical application and normative measurement of ONSD were first established in the 1990s (Hansen and Helmke, 1996). Subsequently, ONSD was widely studied and applied in clinical practice. The measurement of ONSD using ultrasound is preferred because of its low cost, reproducibility, availability, and noninvasiveness. Ultrasound was used to measure ONSD in our study because medical resources and diagnostic capabilities are usually limited in high-altitude environments. A meta-analysis in 2023 pooled six articles on the association between ONSD and AMS symptoms. The majority of studies showed that ONSD was increased during ascent regardless of AMS or not (Lau et al., 2023). In our results, we find that increased ONSD values were observed in both HM and non-HM trekkers from ground to 1,700 m. Our data, although far from conclusive, provide additional information regarding ocular physiological responses at high altitudes.

Research has shown a good correlation between ONSD measured using ultrasound and magnetic resonance imaging (MRI) (Roemer et al., 2023). Nguyen et al. performed MRI to evaluate myopic and emmetropic eyes. They found a smaller cross-sectional area of the optic nerve and subarachnoid space in myopic eyes; however, the overall diameters of the optic nerve sheath complex were similar between the two groups (Nguyen et al., 2021). Our data followed a trend similar to that of Nguyen’s study results. The ONSD values during ascent were not different between HM and non-HM trekkers. Further studies are needed to clarify the association between ONSD and myopia at high altitudes.

### 4.2 Changes in IOP between groups with and without HM exposure in high-altitude environment

During trekking, many physiological and environmental factors can affect the IOP. Exercise contributes to a decreased IOP. Body fluid is shifted to muscle and lost via sweat glands during and after exercise, which results in decreased blood volume and venous pressure and then decreased IOP (Kim and Park, 2019). Bosch et al. (2010a) showed that IOP increased during ascent to 5,533 m and then decreased with further ascent to 6,265 m. They hypothesized that severe hypoxia impairs the function of the non-pigmented ciliary epithelium and thus decreases the production of aqueous humor. Xie et al. (2019) found that hypoxia lowers IOP at high altitudes by decreasing PaCO_2_ and bicarbonate levels as a result of hyperventilation, which contribute to decreased production of aqueous humor. Ersanli et al. (2006) revealed a temporary increase in IOP in 34 healthy male pilots, either under normoxia or hypoxia in a simulated hypobaric chamber. They found that IOP changes were not only related to altitude but also to acclimatization.

In contrast, Xin et al. found that individuals with high myopia presented less configuration changes when the ciliary body contracted (Xin et al., 2020). Thus, a smaller change in the configuration of the ciliary body causes slower regulation in response to the IOP. Xu et al. found that IOP increased after ocular compression and that participants with HM had slower IOP decay (Xu and Lam, 2023). In our study, IOP increased in participants with HM on day 1 afternoon at an altitude of 3,150 m. Although the increase was not significant, HM trekkers had greater IOP levels as compared to non-HM ones at that time point. These findings may be due to the impaired regulation of IOP in highly myopic eyes. As the duration at the high altitude increased, the IOP decreased after acclimatization.

### 4.3 Correlation of changes in ONSD and LLSS

The possible factors affecting ONSD during ascending to altitude include craniospinal and spatial compensations. Spatial compensation accommodates ICP by shifting the CSF from the intracranial space to the spinal thecal and dural sacs. Craniospinal compensation refers to elastic properties of the craniospinal system. Craniospinal systems can buffer CSF fluctuations to prevent large changes in ICP (Lawley et al., 2016). Although transient elevation in ICP could activate trigeminal afferents, which triggers headache (Rolan, 2015), the mechanism of compensation in the CSF tends to maintain ICP in the normal range.

Kanaan et al. (2015) performed a cohort study and found that changes in ONSD were higher in participants with AMS. Currently, no physiological parameters detected at moderate altitude can be useful as a sensitive measurement for early prediction of trekkers suffering from AMS while ascending to high altitude. Our study revealed that fractional changes in ONSD on day 0 at an altitude of 1,700 m correlated with LLSS and headache symptoms on day 2. We chose LLSS data from day 2 because previous studies have shown that AMS severity peaks at that time point (Luks et al., 2017). Additionally, in our study, all participants with AMS had mild symptom severity, which might not have the profound effects of increased cerebral blood flow and cerebral edema. According to the above hypothesis, our results imply that fractional changes in ONSD at moderate altitude might be a predictor of mild AMS.

### 4.4 Correlation of changes in IOP and LLSS

Cerebral edema may result from AMS or HACE (Sagoo et al., 2017). Reliable noninvasive methods for monitoring increased intracranial cerebral pressure (IICP) are desirable for clinical application. IOP has been previously studied for its correlation with ICP. When ICP increases *in vivo*, it can compress the episcleral veins that drain blood from the eyes, resulting in increased resistance to the outflow of aqueous humor and a subsequent increase in IOP. This is known as the “venous stasis” theory, and it suggests that ICP has a direct effect on IOP (Widakowich, 1976; Han et al., 2008; Ghate et al., 2021). Although ICP is correlated with IOP, the variability between individuals is large (Sheeran et al., 2000; Yavin et al., 2014). In our study, IOP did not seem to correlate with the LLSS. A possible reason why IOP was not associated with LLSS was that the symptoms of AMS in our participants were not severe enough to cause IICP. Sheeran et al. found that an increase of 11 mmHg in ICP caused a 1 mmHg rise in IOP (Sheeran et al., 2000). In our study, the severity of AMS was mild, and no participant had symptoms of HACE. Hence, our study showed that fractional changes in IOP were not correlated with the LLSS. A greater increase in ICP is required to achieve a significant elevation in IOP.

### 4.5 Limitations

Our study had some limitations. Although the height of the main peak of Xue Mountain is 3,886 m, we only stayed there for a short period. We returned to 369 Cabin (3,150 m) soon after reaching the peak. Thus, the incidence of AMS was not as high as previous research showed (prevalence of 38% at 3,500 m) (Mairer et al., 2009). The incidence of AMS in our study was 14.8% and most of the participants had mild symptoms. Assessing ONSD as a surrogate measure and marker for AMS is challenging at high altitudes due to various adaptive responses. These adaptations include respiratory alkalosis resulting from compensatory tachypnea, changes in sleep patterns, heightened sympathetic tone, and the ambient hypobaric and hypoxic environment (Strapazzon et al., 2014; Dinsmore et al., 2017). Another limitation arose from the lack of control over the use of acetazolamide due to health and safety considerations. Participants were merely advised to refrain from taking acetazolamide within the 8 h leading up to the ophthalmic examination. Acetazolamide is a reversible carbonic anhydrase inhibitor. It not only increases renal excretion of sodium, potassium, bicarbonate, and water, but also decreases ICP and CSF secretion by acting on the choroid plexus (Uldall et al., 2017). Two participants did not take prophylactic acetazolamide. However, they consumed acetazolamide due to symptoms of AMS during the trek. During ascent, central corneal thickness is also affected by hypobaric hypoxia. Hypoxia leads to a reduction in oxidative metabolism, prompting a shift towards anaerobic glycolysis and subsequent lactate accumulation. The elevated lactate levels, in turn, trigger an influx of water, ultimately resulting in corneal edema (Bosch et al., 2010b). As IOP is influenced by corneal thickness, increased corneal thickness may lead to an overestimation of the IOP (Morris et al., 2007).

## 5 Conclusion

At moderate altitude, fractional changes in ONSD could be a predictor of mild AMS before ascending to high altitude. There seemed to be no difference in ONSD between the HM and non-HM trekkers in this study. Myopia not only causes comorbidities but also may affect physiological accommodation at high altitudes. HM trekkers may have relatively poor regulation of the aqueous humor at high altitudes. Though further studies are needed to clarify these hypotheses.

## Data Availability

The original contributions presented in the study are included in the article/Supplementary Material, further inquiries can be directed to the corresponding author.
